# EFFECTS OF TREADMILL GAIT TRAINING ON BALANCE IN PARKINSON’S PATIENTS AFTER DEEP BRAIN STIMULATION

**DOI:** 10.1590/1413-785220243203e266917

**Published:** 2024-07-22

**Authors:** Viviane Carolina Sales de Andrade, Angelica Castilho Alonso, Natalia Mariana Silva Luna, Fernanda Botta Tarallo Rogatto, Guilherme Carlos Brech, Danilo Sales Bocalini, Júlia Maria D’Andrea Greve

**Affiliations:** 1. Universidade de São Paulo, Faculdade de Medicina FMUSP, Laboratório de Estudo do Movimento, São Paulo, SP, Brazil.; 2. Universidade São Judas Tadeu, Programa de Pos-Graduação em Ciências do Envelhecimento, São Paulo, SP, Brazil.; 3. Universidade Federal do Espírito Santo, Centro de Educação Física e Desporto, Laboratório de Fisiologia e Bioquímica Experimental, Vitoria, ES, Brazil.

**Keywords:** Parkinson Disease, Deep Brain Stimulation, Postural Balance, Neurological Gait Disorders, Neurological Rehabilitation, Doença de Parkinson, Estimulação Cerebral Profunda, Equilíbrio Postural, Transtornos Neurológicos da Marcha, Reabilitação Neurológica

## Abstract

**Objective:**

After deep brain stimulation (DBS), patients with Parkinson’s disease (PD) typically still present significant gait and postural stability problems, and thus additional interventions are needed. In this way, our purpose was evaluate the comparative effectiveness of treadmill training, with and without body weight support, on balance outcomes among patients with PD after DBS.

**Methods:**

Eleven patients with PD that were using bilateral subthalamic nucleus DBS were evaluated using Time Up and Go test (TUG); Berg Balance Scale (BBS) and Static Posturography. In phase 1, all subjects participated in 8-weeks of treadmill training in conjunction with conventional physiotherapy. After six weeks (wash-out), each patient then participated in a subsequent 8-weeks of treadmill training with partial body weight support.

**Results:**

After the phase 1, there were improvements on the cognitive TUG performance (Before: 15.7 ± 1,8 sec; After: 13.7 ± 3.1 sec; p < 0.01) and an increase of anteroposterior and medio-lateral body oscillation with eyes closed. After the phase 2, there were improvements in conventional (Before: 12.3 ± 2.0 sec; After: 10.7 ± 1.7 sec; p < 0.01) and cognitive (Before: 14.6 ± 3.5 sec; After: 12.5 ± 1.6 sec; p < 0.05) TUG performances. There were no significant changes in the Berg Balance Scale following either training protocol.

**Conclusion:**

Both trainings improved static and dynamic balance and had similar results; however, supported treadmill training seemed to be a potentially superior option, as patients tended to feel safer. *Level of Evidence II, therapeutic studies - investigation of treatment outcomes.*

## RESUMO

**Objetivo:**

Mesmo após a estimulação cerebral profunda (ECP), os pacientes com doença de Parkinson (DP) muitas vezes ainda apresentam problemas significativos de marcha e estabilidade postural, e, portanto, intervenções adicionais são necessárias. Avaliar a eficácia comparativa do treinamento em esteira, com e sem suporte de peso corporal, nos resultados de equilíbrio de pacientes com DP após ECP.

**Métodos:**

Onze pacientes com DP em uso de ECP bilateral do núcleo subtalâmico foram avaliados pelos testes Time Up and Go (TUG), escala de equilíbrio de Berg (EEB) e posturografia estática. Na fase 1, todos participaram de oito semanas de treinamento em esteira em conjunto com fisioterapia convencional. Após seis semanas (wash-out), cada paciente participou de oito semanas subsequentes de treinamento em esteira com suporte parcial de peso corporal.

**Resultados:**

Depois da fase 1, houve melhora no desempenho cognitivo do TUG (antes: 15,7 ± 1,8 s; depois: 13,7 ± 3,1 s; p < 0,01) e aumento da oscilação anteroposterior e médio-lateral do corpo com os olhos fechados. Após a fase 2, os resultados do TUG convencional (antes: 12,3 ± 2,0 seg; depois: 10,7 ± 1,7 seg; p < 0,01) e cognitivo (antes: 14,6 ± 3,5 s; depois: 12,5 ± 1,6 s; p < 0,05) demonstraram melhora. Os protocolos de treinamento não causaram mudanças significativas na EEB..

**Conclusão:**

Ambos os treinos melhoraram o equilíbrio estático e dinâmico e tiveram resultados semelhantes; no entanto, o treinamento em esteira com suporte é uma opção potencialmente superior, uma vez que os pacientes tendiam a se sentir mais seguros. Nível de Evidência II, estudos terapêuticos - investigação de resultados de tratamento.

## INTRODUCTION

 Subthalamic nucleus deep brain stimulation (DBS) has been regularly used in Parkinson’s Disease (PD) to reduce the severity of hallmark symptoms such as bradykinesia, ^1^ rigidity and tremor, ^2^ ^,^ ^3^ however, its effect on postural instability and gait disorders (axial symptoms) is less well-understood. ^1^ ^,^ ^4^

 Gait disorders and balance dysfunction in PD increases risk and frequency of falls, ^5^ ^,^ ^6^ and both parameters worsen during dual task (motor or cognitive) demands. ^7^ Among aging adults with PD, falls can lead to incapacity, morbidity, reduction on quality of life, and even early mortality. ^8^ ^,^ ^9^ Because DBS interventions have uncertain results on axial symptoms and the progressive aspects of the disease, it is important to explore potential adjuvant therapeutic approaches that may provide adaptations for maintaining or even improvements of surgical results in such abilities. 

 Standard physiotherapy interventions are well-known to improve strength, the range of motion, balance, and gait in patients with PD. ^6^ ^,^ ^9^ Moreover, treadmill training with ^9^ ^-^ ^12^ or without body weight support may be an alternative strategy to improve the axial symptoms of PD ^13^ ^-^ ^20^ lthough treadmill unsupported training is certainly more biomechanically specific to free-living ambulatory conditions, the supported training may provide an advantage by allowing for better a perception of safety and comfort, and thus greater progression in training dose (e.g., treadmill velocity, incline, etc.). 

 Moreover, Luna et al. ^21^ conclude that the body weight supported treadmill training promote significative changes in kinematics variables of gait. Therefore, the purpose of this study was to evaluate the benefits of treadmill training and the comparative effectiveness of treadmill training with and without body weight support on mobility and balance in patients with PD after DBS. 

## MATERIALS AND METHODS

### Study design

This study is a Prospective, longitudinal, controlled study. The study was approved by ethics committee of Clinical Hospital, School of Medicine, University of Sao Paulo under number 0105/10, and all the participants have signed statements of informed consent.

### Participants

 Patients with idiopathic PD that were using subthalamic nucleus bilateral DBS were recruited for this investigation. Inclusion criteria were: (1) ≥ 12 months post-surgery, (2) an ability to walk 10 meters without assistance, (3) PD disease stage II-III according to Hoehn & Yahr classification, ^22^ (4) a Mini-Mental State Examination (MMSE) score ≥ 25, (5) stability of medications and DBS parameters, (6) no history of treadmill training in the previous six months, (7) no concomitant physiotherapy interventions, and (8) no other existing neurological disorders. Moreover, patients were excluded if they were not able to perform the evaluations. Of the twenty-nine patients that met inclusion criteria, only 17 were able to participate. Among these patients, six were excluded (2 changed DBS devices, 3 for excessive absence, and 1 for not finishing the evaluation due to a freezing episode). Eleven patients finished protocol, six men and five women. As for the Hoehn and Yahr Classification: ^22^ one patient was classified as 3, seven as 2,5 and three as 2. At Table 1 are described the sample characteristics. 

**Table 1.** Sample characteristics.

Parameters	Mean ± DP
Age (years)	61 ± 2
H&Y	2 ± 1
MMSE	27 ± 1
Diagnostic (years)	20 ± 7
Time after DBS (months)	20 ± 4

### Procedures

**Figure 1.**
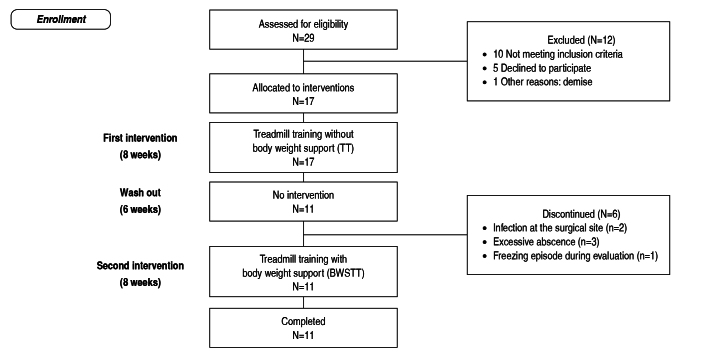
Enrollment and retention of study participants.

 Included patients were submitted to functional tests one hour after taking the medication and with the DBS dispositive active. The evaluation was applied pre and post unsupported treadmill training as well as pre and post body weight supported treadmill training, by the same examiner. The Figure 1 present all phases of study. 

### Intervention

Patients underwent two different treatment phases:

### Phase 1

Phase I consisted of treadmill training without body weight support or body belt. It was conducted in conjunction with a standard physiotherapy intervention that involved stretching, strengthening and balance exercises. Training took place twice per week for a duration of eight weeks (i.e., 16 total sessions). Each session lasted 90 minutes. Treadmill training lasted 30 minutes, in which the initial speed was determined individually for each patient coincident at a comfortable pace, and then gradually increased as each patient improved gait performance. During the bout of walking, patients were carefully monitored for fatigue. In the event that a patient complained of fatigue or any symptom related to overexertion (e.g., shortness of breath, etc.), walking speed was gradually reduced to a comfortable pace. Patients were oriented to hold on treadmill lateral or anterior bars, but during the training, they were encouraged to release hands and increase step length. After phase 1, each patient was re-evaluated on all mobility and balance assessments.

### Wash out

Following the first phase of training, all patients underwent six weeks of wash out. During this period, they were instructed to not participate in any type of physical exercise. The third evaluation of all mobility and balance measures was administered after the washout and before Phase 2.

### Phase 2

During phase 2, each patient participated in treadmill training with body weight support (BWST), which was again in conjunction with the same physiotherapy program as during phase 1. At the beginning of phase 2, BWST was administered at 30% of body mass support and thereafter was reduced to 20% by the sixth session, and to 10% by the eleventh session. The BWST required the use of a body belt with straps to allow body suspension. The number of sessions, training time, and all exercises were similar to that of phase 1.

## EVALUATED PARAMETERS

### Berg balance scale

 The Berg balance scale (BBS) is a balance evaluation, and is comprised of 14 items performed during sitting, standing, and postural transitions. ^23^ The scale is scored from 0 (unable to perform) to 4 (normal performance) ^24^ and has documented high inter-reliability and internal consistency among patients with PD. ^23^

### Time up and go test

 The time up and go test (TUG) measures the time it takes for the individual to get up from a chair to a standing height, walk three meters, return to the chair, and sit back down. This test has a high test-retest reliability and inter-rater reliability in the PD population. ^25^ Patients were instructed to carry out the task at their normal movement speed. The test was applied in three situations: (1) conventional (i.e., the standard test); (2) cognitive – dual task (i.e., the standard test plus answering subtraction calculations); and (3) motor – dual task (i.e., the standard test carrying a tray with a glass of water), as previously described. ^25^ Each test was completed three times and the average times were calculated for the analysis. 

### Static posturography

The postural balance assessment (posturography) was performed on a portable force platform (AccuSway Plus, AMTI, MA, USA). For data acquisition, the force platform was connected to a signal-amplifying interface box (PJB-101) that was linked to a computer by means of an RS-232 cable. The data were gathered and stored using Balance Clinic software, configured to a frequency of 100 Hz with a fourth-order Butterworth filter and a cutoff frequency of 10 Hz. All subjects underwent the test with standardized positioning in relation to the maximum width of the support base (smaller than hip width), with arms along the body and head facing a target. The base of support was drawn on a paper on a fixed position on the force platform, corresponding to the anatomical points of distal hallux phalanx, fifth metatarsal head, and lateral and medial malleolus for each foot. Three measurements were made with the eyes open (EO) and three made with the eyes closed (EC) for 60 s each. The arithmetic means of the results were calculated from the three tests conducted under each condition and were processed using the Balance Clinic software. The parameters used to measure the subjects’ stability with eyes open and closed were the root mean square of the displacement amplitude from the COP in the mediolateral axis (XSD), anteroposterior (YSD) and the mean velocity calculated from the total displacement of the COP in all directions (VAvg).

### Statistical analysis

The data were described by medians, means, and standard deviation. The Shapiro-Wilk test was used to determine whether the continuous variables presented a normal distribution. The following comparisons were made: (1) pre versus post unsupported treadmill training evaluation (i.e., phase 1), (2) before versus after BWST evaluation (i.e., phase 2), and comparisons between phases for absolute and relative (%) changes in outcomes. Student t-Test was used for the comparison of TUGT and Berg Balance Scale results. Regression analysis with post-intervention outcomes as the dependent variable and baseline values as covariates were also used to assess the between-group differences in TUGT and Berg tests. The models included a group indicator with two levels and baseline values as covariates. This model is equivalent to an analysis of covariance (ANCOVA) but has the advantage of providing estimates for each group, adjusted for baseline characteristics that are potentially associated with the primary outcomes. A coefficient of the unsupported treadmill group indicator was employed to estimate the mean post-intervention outcome (e.g. conventional TUG) associated with unsupported treadmill, compared with BWST. Regression assumptions were checked. Posturography variables were analyzed using the Wilcoxon Test. All data were stored and analyzed on SPSS v20.0 and statistical significance was set at p < 0.05.

## RESULTS

 The conventional TUG decreased significantly from baseline, following the BWST, but no differences were seen after unsupported treadmill training ( Table 2 ). Despite the pre to post-intervention differences, after adjusting for baseline values there were no differences between phases for post-intervention values (p > 0.05); however, there was a non-significant trend of greater improvements in conventional TUG for BWST (11.9%) as compared to unsupported training (3.4%) (p = 0.08). The cognitive TUG decreased significantly following both unsupported treadmill training (13.1%; p = 0.01) and BWST (11%; p = 0.04), but there were no differences between phases after adjusting for baseline values (p = 0.48). There were no changes in the performance of the motor TUG following either intervention and no differences between phases. Berg Balance Scale results demonstrated no differences from pre and post-intervention for unsupported treadmill training and BWST, nor when comparing interventions. 

 Static Posturography evaluation with opened eyes showed no differences to anterior-posterior and mediolateral amplitude, or in velocity of the pressure center displacement following unsupported treadmill training and BWST ( Table 3 ). During the closed eyes evaluation, the anterior-posterior and medio-lateral amplitude increased after the unsupported treadmill training, but there were no changes in the velocity of the pressure center displacement. There were no differences in any outcomes following the BWST, and there were no differences between treatments. 

**Table 2.** Conventional, cognitive and motor Time Up and Go test and Berg balance scale outcome measures before and after the treadmill training without and with body weight support in Parkinson’s disease patients using deep brain stimulation device.

Parameters	Phase 1 Treadmill training without body weight support			Phase 2 Body weighted supported treadmill training			After Phase 1 vs Phase 2
	Before	After	P ^*^	Before	After	P ^*^	P ^*^
Conventional TUG (sec)	11.8 ± 2.1	11.4 ± 2.6	< 0.25	12.3 ± 2.0	10.7 ± 1.7	< 0.01	< 0.28
Cognitive TUG (sec)	15.7 ± 1.8	13.7 ± 3.1	< 0.01 ^*^	14.6 ± 3.5	12.5 ± 1.6	< 0.05	< 0.26
Motor TUG (sec)	13.9 ± 3.0	13.3 ± 3.8	< 0.19	13.3 ± 3.0	11.9 ± 1.6	< 0.14	< 0.15
Berg balance scale	50.7 ± 3.0	51.9 ± 3.1	< 0.22	50.0 ± 4.4	51.7 ± 1.6	< 0.18	< 0.83

* Test t student

Values expressed in mean ± DP. TUG: time up and go.

**Table 3.** Static posturography parameters with opened and closed eyes evaluation before and after the treadmill training without and with body weight support.

Parameters	Phase 1 Treadmill training without body weight support			Phase 2 Body weighted supported treadmill training			After Phase 1 vs Phase 2
	Before	After	P ^*^	Before	After	P ^*^	P ^*^
Opened eyes							
Med. lateral amp (cm)	1.34	1.54	< 0.13	2.24	2.43	< 0.79	< 0.24
Ant. posterior amp (cm)	1.60	3.24	< 0.11	2.98	2.75	< 0.79	< 0.47
Mean velocity (cm/s)	0.70	0.98	< 0.37	1.04	1.01	< 0.37	< 0.42
Closed eyes							
Med. lateral amp (cm)	1.38	3.28	< 0.01 ^*^	2.33	2.07	< 0.72	< 0.42
Ant. posterior amp (cm)	2.15	3.71	< 0.04	3.22	3.16	< 0.79	< 0.47
Mean velocity (cm/s)	1.21	1.22	< 0.18	1.24	1.20	< 0.53	< 0.59

* Wilcoxon Test.

Values expressed in median. Med. lateral amp: medio lateral amplitude of dislocation of the pressure center in centimeter. Ant. posterior amp: anteroposterior amplitude of dislocation of the pressure center in centimeters

## DISCUSSION

 Treadmill training could also be associated with neuroplasticity and neuroprotection, as experimental animal studies have previously demonstrated. ^26^ The unsupported treadmill training improved cognitive TUG and improved certain aspects of static posturography, i.e., anterior-posterior and medial-lateral amplitude of dislocation of the pressure center with eyes closed, but not with eyes opened. Body weight supported treadmill training improved conventional and cognitive TUG performance; however, it did not promote changes in posturography outcomes. Likewise, outcomes from the Berg Balance Scale were not changed from either intervention. 

 Performance in the TUG is highly correlated to mobility, the risk of fall and severity of the PD symptoms, ^27^ and is moderately correlated to gait velocity. ^28^ Each second increase in the TUG is associated with a 2.3% increase in the odds of a fall. ^29^ We have demonstrated that treadmill gait training both with and without body weight support can improve performance in the TUG among persons with PD, and these improvements could be related to the increase in gait velocity and step length promoted by the direct effect of the treadmill. ^29^ ^,^ ^30^

 A reduction of TUG could also be associated with a balance and motor control improvement, facilitating the execution of the movement and the preparation to turn and sitting to stand. ^30^ Some experts have described such a reduction in conventional TUG after unsupported treadmill training, ^14^ ^,^ ^19^ ^,^ ^31^ however, we demonstrated this only in conjunction with BWST. The absence of differences after unsupported treadmill training may have been due to less training sessions per week, less duration of treadmill training sessions in comparison to previous studies, and/or a higher pre-training functional status due to the DBS treatment among our patients. 

The reductions in conventional TUG were seen only after the body weight support treadmill training, which could be due to the increase of the gait velocity and step length in this particular kind of training, thus making this a more effective strategy than the unsupported treadmill training. It is also possible that because body weight support treadmill training provides more safety and confidence, patients can release hands earlier in the intervention and reinforce balance recruitment. These factors could contribute to the long-term effectiveness of the treadmill gait training in PD.

 Cognitive TUG decreased after both training protocols, which could be associated with an improvement in gait automaticity. Dual-task affects PD gait performance reducing velocity, step length, swing time and increasing double stance due to a competition for neurological available resources. ^32^ ^,^ ^33^ Treadmill gait training, particularly with body weight support, can elicit improvements in balance and mobility, demanding less attentional resources during these tasks and probably facilitating cognitive requirement and motor performance. 

 Despite being a dual task, motor TUG did not change following either type of training. The dual motor task could be easier than the cognitive task because involves two motor tasks demanding lower attentional sources. ^34^

 Previous studies have shown improvements in BBS after treadmill training without and with body weight support. ^10^ ^,^ ^13^ However, the present study did not find the same results and this could be due to a higher baseline BBS capacity among our patients. Our small sample size is another potential factor limiting the ability to detect significant differences in BBS parameters. ^10^ ^,^ ^15^

 Following the unsupported treadmill training, the oscillation of the pressure center (COP) increased with closed eyes. The increase of the anterior-posterior and medial-lateral amplitude of dislocation of the COP with eyes closed could suggest the increase of the limits of stability. Some previous work, however, has described this increase in body oscillation as a worsening in balance, with a higher risk of fall. ^35^ ^-^ ^39^ However, these findings are controversial in PD, because as patients have a rigidity and flexed posture, this decreased the COP oscillation. ^40^

 The reduction of the compensatory dynamic postural responses, as well as the range of motion of the COP, could lead to a loss of balance during dynamic conditions. ^36^ Moreover, the increase of the COP amplitude oscillation, seen after treadmill training may well be associated with improvements in balance, as it means a large limit of stability that could be favorable on daily activities as well as in regards to reduction of falls risk. 

Despite not observing differences between training protocols, the use of the body belt promoted more safety and it allowed patients to release hands from handrails, thus increasing balance stimulus during the BWST. This type of treadmill training appears to be more effective among patients that require greater assistance.

Some limitations should be mentioned. First, we had a very small sample to detect multiple outcomes from various tests. Future, prospective studies with larger sample sizes are necessary to better understand the trajectory of changes between unsupported and supported treadmill training. Moreover, and despite the 6-week wash-out period, there may have been a training effect from using unsupported prior to supported training for all subjects. It would be interesting for future efforts to examine these in a random assignment, as well as to examine the effectiveness of varying doses of the treatment. Despite these limitations, we provide some of the very first evidence to document the comparative effectiveness of supported versus unsupported treadmill training among patients with PD, following DBS therapy.

## CONCLUSION

Treadmill training and body weight supported treadmill training is safe and effective strategies to improve balance and mobility among patients with PD; however, patients seem to feel more confidence during body weight supported treadmill training. Both types of training can be used as an adjuvant treatment of the DBS surgical procedure, for improving physical capability, balance and gait stability and reducing the risk of fall.
